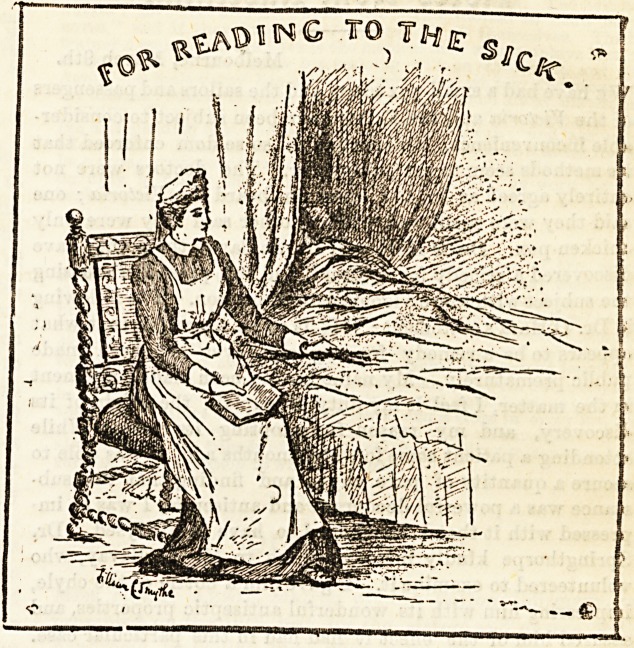# The Hospital Nursing Supplement

**Published:** 1891-05-02

**Authors:** 


### "The Hospital" Nursing Mirror.

Being the Extra Nursing Supplement of "The Hospital" Newspaper.

Contributions for this Supplement should be addressed to the Editor, The Hospital, 140, Strand, London, W.C., and should have the word "Nursing" plainly written in left-hand top corner of the envelope.

### En Passant.

**SHORT ITEMS.**—Mrs. Phillips, of Norwood Cottage Hospital, writes to state finally that her niece, Nurse Phillips, resigned her post at Paddington Green Children's Hospital of her own accord.—A Gentleman's Committee has been formed at Basingstoke to keep on the services of the Roman Catholic nurse dismissed by the Ladies' Committee. —The Brassey Holiday Home reports 250 visitors for the year.—The Matron of Poplar Hospital has resigned; the Assistant Matron of Kingston Infirmary has resigned.—The South Wimbledon District Nursing Association cared for 132 cases last year; the nurse is Miss Hagnerim.—A branch of the Queen Victoria Institute has been started at Inverness, Lady Frances Baillie is President.—Mrs. St. A. Horton has opened a nursing and convalescent home at Richmond House, Worthing.—Miss M'Callum, the district nurse at Ayr, visited 326 patients last year.—The Duchess of Teck will present the medals to the Mary Adelaide nurses on May 14th, at the Conduit Street Galleries.

**FIGURES FOR NURSES TO NOTE AND REMEMBER.**—The nursing controversy has come to a head. We publish elsewhere an important letter from the Nurse's Training School authorities and also Miss Nightingale's opinion. As the Treasurer of St. Thomas's Hospital showed in our columns last week, the present unfortunate state of disagreement has been brought about by the action of the Royal British Nurses' Association, which has placed itself in direct opposition to the expressed opinion of nearly all those who know most of the subject. The report of the Royal British Nurses' Association recently issued with the accounts for the year ended June 30th, 1890, proves that Mr. Wainwright's view is shared by doctors, matrons, and nurses alike. This will be seen at a glance by comparing the numbers of the three classes of members as given in the accounts for the periods and year ended June 30th, 1889 and 1890 respectively. The following table brings out the facts clearly :—

| Period or Year ended 30th June. | Life Members. Matrons and Sisters. | Life Members. Nurses. | Doctors and Matrons at 10s. 6d. per annum each. | Nurses at 2s. 6d. per annum each. | Total Members. |
|---|---|---|---|---|---|
| 1889 | 28 | 230 | 452 | 2,746 | 3,456 |
| 1890 | 3 | 111 | 170 | 1,687 | 1971 |
| **Decrease in 1890** | ... | ... | 282 | 1,059 | 1,341 |

It thus appears that the total number of members who have paid their subscriptions, and may therefore be properly deemed to belong still to the Royal British Nurses' Association, according to the last report, is 1,857 only, to which we must add 372 life members, making in all but 2,229 members, if doctors, matrons, sisters, and nurses are all included. What are we to say, then, to the statement so often made that upwards of 3,000 nurses are members of the Royal British Nurses' Association? The Hon. Secretary, it is true, declared at the annual meeting that once a member of the British Nurses' Association always a member, whether you pay your subscription or not, so long as you retain the card. This is a novel doctrine, and as a calculation it is entirely upset by the large number of old members who are reported to have burnt their cards. Besides, when the members who pay their subscriptions to an association decrease from 3,198 to 1,857, the present number as shown by the last published accounts—that is to say, by 1,341 in one year—is it not fair to conclude that its objects and methods do not commend themselves to those for whose benefit it was ostensibly established? At this rate the Association must soon die of inanition!

**TORQUAY INSTITUTE.**—The fourteenth annual report of this Institute showed 7,646 district visits made during the year. The staff is now increased to 22; the expenditure last year was £979. The Committee have joined the National Pension Fund, and a friend has kindly offered to give £5 if nine others will do the same before Easter, 1891. The Committee have granted a small pension to two nurses, who have faithfully served the Institute for many years, and who are too old to join the Pension Fund. A little leaflet is pasted outside the report calling attention to the fact that grateful patients can subscribe to the R.N.P.F.

**SERVICE FOR THE KING.**—This is the title of the Mildmay Magazine, which tells of all the work done by the deaconesses. The Deaconess House adjoins the Conference Hall at Mildmay Park, and about fifty ladies are resident; the Lady Superintendent is Miss Coventry. The workers for South London are most resident at the Deaconess House, Effra Road, Brixton, where Mrs. McNaughten is in charge. Many outlying districts of London are looked after, also institutions at Brighton, Northampton, Oxford, and, going still further afield, at Jamaica, Malta, and Jaffa. The new hospital at Bethnal Green will be occupied this year; 346 in-patients were treated last year in the old building. The Barnet Convalescent Home received 609 patients last year. Our readers must be well acquainted with the quaint uniform of the Mildmay nurses, who are sent out to private cases from the Nursing House at Newington Green.

**YESTERDAY AND TO-DAY.**—The nurses on the staff of King's Cross Hospital, Dundee, are very indignant at the tone a local paper uses in speaking of nurses. They send us this cutting: "Talking of riddles, I was asked to-day by a person deeply interested in the social welfare of the community—Why do nurses and washerwomen, almost without exception, insist upon being served with spirits? I am informed that it is next to impossible to conduct a home on temperance principles and obtain the services of this class of females, and that their employment necessitates strong drink being brought into houses where otherwise it would be unknown. If this be so, it is an evil which ought to be put an end to by some means or other." The expression "nurses and washerwomen" gives us much offence as the more serious imputation; but we beg our Scotch cousins to regard the paragraph as a joke; such rubbish is not worth serious attention.

**HOLIDAYS.**—Many a nurse is now planning how to spend her precious summer holiday, so perhaps a few suggestions may be valuable. A fortnight at St. Malo can be managed for £8; trips can be made to Mont St. Michel, Dinan, and other pretty spots in the neighbourhood; the Hotel de France at St. Malo is very comfortable, and not dear. Seaside holidays can be spent at the Brassey Home, St. Leonards, where the charge is from 15s. a week; or with Sister Dora at Scarborough. Then two ladies, who have a farm in Essex, have laid themselves out this summer to benefiting nurses by giving them a real country rest. Russell's Farm, Wethersfield, Braintree, is the address, and there is unlimited milk and fresh eggs, and a pony-trap to drive about the pretty country lanes. Only no nurse must go to Wethersfield after being at an infectious case, for it is a dairy farm. Wales and Scotland offer unlimited opportunities for holidays, and perhaps it is worth while to state that the Temperance Hotel at Inverness is cheap and comfortable, and makes good head-quarters for a Scotch tour. Two nurses spent a fortnight in Holland last year, cost £10; other two got as far as the Rhine. These are mere suggestions—nurses are not so helpless as to need their trains pointed out, or their probable expenses estimated for them—we leave the details to others.

### Lectures on Surgical Ward Work and Nursing.

By Alexander Miles, M.B. (Edin.), C.M., F.R.C.S.E.

LECTURE XXII.—SCOTT'S DRESSING.

Uses.—This application is used in the treatment of chronic joint disease, especially of a tubercular nature. It combines the advantages of the application of mercury and of pressure, both acting in the direction of promoting absorption of the morbid products. At the same time it gives a certain amount of fixity to the joint, thus securing rest, but this action must be augmented by the use of some form of splint if it be deemed an important part of the treatment.

Materials Required.—(1) A quantity of plain white lint; (2) a quantity of blue ointment (mercuric ointment); (3) a quantity of strong moleskin plaster; (4) domette bandage; (5) spatula; (6) scissors; (7) safety pins; (8) sheet of brown paper; (9) means of heating the plaster.

Method of Preparing the Material.—Cut a piece of lint of sufficient size to extend all round the affected joint, and for an inch or two above and below it. Shape the lint so that when applied it will lie smothly without overlapping. Spread out the brown paper on a flat table, and then with the spatula lay on the lint a pretty thick and even layer of the mercuric ointment. The brown paper is to prevent soiling of the table as you cover the edges of the lint with the ointment. It is immaterial on which side of the lint the ointment is spread; as a rule it is more evenly spread on the plain side. Next cut about twelve or fourteen strips of adhesive plaster, measuring in length about one and a quarter time the circumference of the limb, and in breadth about one inch; and one piece of the same breadth, but long enough to go twice round the limb at the upper level of the lint.

Method of Applying the Dressing.—Having washed the whole limb with soap and water and turpentine, examine it carefully to see that there are no scratches or open sores, as by these the ointment may be absorbed too rapidly and lead to symptoms of mercurial poisoning. Any scratch should be covered with some wool and friar's balsam, or collodion. Of course a joint in which sinuses exist is not suitable for Scott's dressing, and so the question as to the method of dealing with these does not arise. Next apply the lint which you have already prepared, making it lie as smoothly as possible. The limb is then placed in the position which it is desired to retain while the dressing is on. This will be indicated to you by the surgeon. The plaster strapping has now to be applied. Having heated a strip, by placing its non-adhesive side against a jar containing very hot water, or otherwise, pass it round the joint so that the two ends cross in the middle line of the leg in front. The first strip should be at the lowest level of the lint, and each succeeding layer overlaps two-thirds of its predecessor. All the crossings should be kept in the middle line, and the ends should go about an inch and a-half beyond the point of crossing, all being cut to the same length. In applying the plaster the pressure should be directed from below upwards. When the upper limit of the lint is reached, the long strip of plaster which you have prepared should be wound round the limb so as to cover in the loose ends at the top, and so give the dressing a neat appearance. The whole dressing is now completed by a divergent spica bandage. If necessary an appropriate splint shall be applied over all.

After Treatment.—Keep a careful look-out on the toes to guard against undue pressure and interference with the venous return. Should the patient complain of it being too tight, the edges may be snipped with scissors to relieve it a little. After a fortnight or so the whole dressing will usually be found to have become quite slack and untidy. This is partly due to the movement of the limb, but mainly to the decrease of the swelling on account of the absorption of the diseased material. It should then be removed, the joint washed, and, if necessary, the dressing reapplied.

### Miss Nightingale and the Registration of Nurses.

At a meeting of the representatives of hospitals and training schools recently held at St. Thomas's Hospital, Mr Rathbone, M.P., speaking on behalf of the Nightingale Training School for Nurses, against the recognition by the Board of Trade of the establishment of a registry of trained nurses by the Royal British Nurses' Association, said:—

I regret extremely that we are deprived to-day of the presence of Mr. Bonham-Carter, who has been, from soon after its foundation, the Secretary of the Nightingale School, the parent of so many of the nursing training schools throughout the kingdom, and of the reform of hospital nursing. It is at his request, and in accordance with the wish of Miss Nightingale, that I attend here to-day, as one of the Executive Committee of the Training School of the Nightingale Fund, to say in Miss Nightingale's own words, that she does "not think that a system of registration such as that proposed, is for the benefit of the nurses." It would be most unfortunate if personal antagonisms and rival claims should be mixed up with the vital question as to a system of registration, such as that adopted by the British Nurses' Association, being for the benefit either of the nurses themselves, or of the public. There seems an almost complete consensus of opinion on the part of all those who have done most of the training and improvement of nurses during the last thirty years, that the scheme of a general register would tend to confuse and mislead the public, and, so far from promoting the improvement of nursing in this kingdom, that it would tend to check its further progress; nay, more, that it would tend seriously to lower the standard which we have to some extent attained, and to stereotype a standard of nursing dangerously low for both rich and poor. Such results could not be otherwise than injurious to the interests of both nurses and the public. It is certain that the plan proposed will recommend to the confidence of the public a great number of utterly untrustworthy, incompetent nurses, and it will be practically very difficult to take their names off the register, though they may have proved themselves unworthy of such confidence. If it be asked, what alternative have we to propose in order to protect the public from incompetent nurses, and guarantee to them efficient nurses, I should reply, that it would require the most careful consideration, and the combined wisdom of those who have been so long at work in raising the standard of nursing and the supply to the public of good nurses, to contrive and organise a safe alternative. It is only about thirty years since the public, or even the hospital authorities, became alive to the necessity of thoroughly trained nurses. The number of good, well-trained nurses is increasing rapidly, but they are still very few, in comparison to the large majority who are imperfectly trained or incompetent. As Miss Nightingale has said, "twenty or thirty years hence, when so much progress has been made, that our present time is looked back upon as the time of bad nursing, this registration might do." In a recent letter to me on the subject, Miss Nightingale says, "You cannot select the good from the inferior nurses by any test or system of examination. But most of all, and first of all, must their moral qualifications be made to stand preeminent in estimation. All this can only be secured by the current supervision, tests, or examinations, which they receive in their training school or hospital, not by any examination from "a foreign body" like that proposed by the British Nurses' Association. Indeed, those who came off best in such would probably be the ready and forward, not the best nurses." It is not only that those experienced in nursing work consider the proposal dangerous, but I believe it is without precedent to give public, official recognition to a body like this, who have no successful experience to produce against the protest of the great body of those who have been most instrumental in raising the standard of nursing and improving and elevating the position of nurses.

The chief nurse-training schools throughout the country have already endorsed Miss Nightingale's protest, and a Committee of their representatives is now sitting at St. Thomas's Hospital, under the presidency of Mr. Wainwright, the Treasurer.

### Nursing Medals and Certificates.

### ST. BARTHOLOMEW'S HOSPITAL.

ST. BARTHOLOMEW'S is one of the largest and best known of the training schools for nurses; lectures are delivered and half-yearly examinations held, and prizes in books varying in value from £1 11s. 6d. to £5 are given yearly. But the chief honour for which the Bart's nurses strive is the gold medal pictured above which is awarded every April and October to the probationer who passes first in the final examination. This medal was first instituted in 1885, when it was won by Nurse Turner; in 1886 it was won by Nurse Shrives and Nurse Constable; in 1887 by Nurse M. G. Smith, Nurse Snedley, and Nurse Freeman; the first two being bracketed equal for the spring examination. In 1888 it was won by Nurse Wisden and Nurse Stevens; in 1889 by Nurse Hunter and Nurse Eames. Last year four medals were given, for at the April examination Nurse Wilson and Nurse Megginson proved equal, and at the October examination Nurses Harrison and Waind were bracketed together. The St. Bartholomew's certificate is given at the end of three years.

### CAMBORNE DISTRICT NURSING ASSOCIATION.

### LIFE'S EDUCATION.

OUR life is given us by God for many different purposes, chiefly as a preparation for an eternity with Him, therefore we must keep ourselves free from selfishness and unkind thoughts and actions towards our neighbours; distinctly we are not to consider our own ease and pleasure the great end of our existence.

If we do not try to educate ourselves God sends sickness, pains of body and mind to recall us to a sense of duty. The lessons we learn in this way are seldom pleasant and often very hard, but if we get them by heart, one by one as they come, they seem easier and easier till at last we bless Him who has taken such pains to fit us for our future life.

And that sorrow and suffering does improve our characters there is no doubt. From suffering we learn patience, and the loss of those most dear to us makes us draw nearer to One who is dearer than any earthly friend or brother. Money failures and poverty show us that our daily bread comes from a higher power which only blesses our efforts when they are in a right direction. An ill temper with which we have to contend makes us pity one who is a curse to himself as well as all around him.

Education is the drawing us to higher and better things, Insensibly we leave behind us the petty cares and wishes of a foolish and aimless life. We must never think we have finished our education, as the saying goes; we are never too old to learn, nor is it ever too late to mend. Education ends only with our life. The nearer we get to the last days the more genial and sympathetic we shall grow to our fellow creatures and patient, humble, and meek with what is against the grain to ourselves.

We must not forget that it is the Master alone who can teach properly, and we must not seek after false teachers who will only lead us astray. We should go to Him and say—

Father, I know that all my life
Is portioned out for me,

Wherever in the world I am,
In whatso'er estate,
I have a fellowship with hearts
To keep and cultivate;
And a work of lowly love to do
For the Lord on whom I wait.

### Notes from Australia.

Melbourne, March 9th.

We have had a small-pox scare, and the sailors and passengers of the *Victoria* and the *Kelton* have been subject to considerable inconvenience. Quarantine is so seldom enforced that its methods seem to get out of gear. The doctors were not entirely agreed as to the cases on board the *Victoria;* one said they were small-pox, while another said they were only chicken-pox. Dr. O'Hara and Dr. de Bavay think they have discovered a remedy for typhoid, and the press is discussing the subject with more vigour than discretion. The following is Dr. O'Hara's statement: "It is to be regretted that what appears to be a remedy for typhoid fever has been made public prematurely. My name having been made prominent in the matter, I feel it my duty to explain the origin of its discovery, and my reason for adopting its use. While attending a patient some fourteen months ago, I was able to secure a quantity of pure chyle, and finding that this substance was a powerful deodorant and antiseptic, I was so impressed with it that I determined to have it analysed. Dr. Springthorpe kindly introduced me to M. de Bavay, who volunteered to examine it. I gave him a bottle of the chyle, impressing him with its wonderful antiseptic properties, and assured him of the effect it had had in this particular case. M. de Bavay, after careful investigation, found in the fluid an organism which when cultivated proved highly destructive to the bacillus of typhoid. Having then at my disposal a harmless substance containing a germ-destroying agent, I tried it in a variety of diseases which depend for their existence upon germs. I can only say that so far my success has been complete. My friend and colleague, Dr. C. L. Lempriere, at my request is giving it a trial at the Alfred Hospital, and will give his opinion later on. Other well-known medical men are also trying it with most satisfactory results. So far, then, everything has been favourable, but time alone will prove the value of the remedy." Dr. Anderson, of the Alfred Hospital, stated that the remedy had been applied so far in five cases, but it would be difficult to say that any decided result had followed. In three cases it was applied on the 13th of the present month, one of them being a bad case, certainly, but by no means a case of which it could be said that a recovery would not have taken place apart from any treatment. The temperature chart in this particular case shows that immediately following upon the administration of the remedy the patient's temperature began to come down, and continued to fall for a time, when it suddenly and unexplainably rose again, and just as suddenly fell to something pretty nearly the normal temperature. Because a certain result followed the application in this particular case, however, it by no means followed that the result must come from a particular cause. In the other two cases where the remedy had been applied on the 13th of this month it did not appear to have interfered with the course of the disease in any way, the cases being in neither instance severe ones, while the patient was in each case progressing favourably before the remedy was tried. In the other two cases completing the five in which a trial was being made, the curative germs had only been administered a day or two since, and it was impossible yet to say anything as to results. The method of application has so far in the case of typhoid patients been to place some of the organisms in all the food given to them, as it has been satisfactorily shown that it may be so administered without injury.

The Salvation Army work here is being subjected to adverse criticism, especially in some of the evidence before the Charities Commission. During February the Commissioners visited Geelong, Beechworth, Kilmore, Wangaratta, and other local hospitals and institutions: they found in some a total absence of any protection from fire. There were also faults in the administration of many of the hospitals; some of these up-country institutions are anything but perfect.

Two Melbourne doctors have been to Berlin, and their reports on Koch's lymph have been printed in full in the *Argus.* We are not quite such sticklers for etiquette here as at home.

On February 19th, the Governor, Lord Hopetoun, opened the Maroondah River Water Trust, a valuable addition to the Melbourne water supply. The river runs through forty miles of lovely country, the gullies being rich in tree ferns and beautiful foliage.

The way this City of Melbourne grows in spite of everything that can be done to stop it would surprise an Englishman. We never cease building, and our buildings are second to nothing I have seen in London. This morning, March 9th, I passed a bank with windows and balconies that are simply full of Ruskin and Venice, went on by other noble structures, turned up William Street, admired our splendid Law Courts, and then found myself at Mr. Fitzgerald's private hospital. It was commenced some time since by Miss Farquharson, but is now in the hands of Mr. Fitzgerald. The Lady Superintendent is Madame Perrin. The house is a three-storied one, with balconies and verandahs and a narrow strip of green garden in front, refreshing to look at when the thermometer marks 90 in the shade. Eighteen patients can be taken, and these have to pay for their rooms alone from £5 to £7 each per week. There are ten nurses, each receiving £45 per annum. Every other day the nurses have holiday from 2 o'clock to 5, and every other evening from 6.30 to 10; and once a month, the whole day. Mr. Fitzgerald, usually styled Doctor, has a great reputation as an operator, and deserves it. He has many very difficult cases accordingly, and these can be better treated in a private hospital than at home. Home often means living at a "station"; some of these are 50 miles square, and the nearest doctor 70 from the station, in some cases more than that. So the squatter prefers to come to the City when he needs scientific nursing and the best surgery. "He gets jarred sometimes," said the nurse I saw. The other day a squatter's widow was here for a slight operation; when she went away she offered me five shillings. I could only say, "When one lady does for another what I have done for you, she does not expect to receive money, thank you, I would rather not." Happily such want of tact does not often occur, and our work is usually very interesting.

### GRAFTON COLLEGE.

This institution has been recently started at 35, Fitzroy Square by Mr. H. Newman Lawrence, M.I.E.E., and his colleague, Dr. Arthur Harries. It forms a fresh evidence of the energy of these gentlemen in their efforts to place in a satisfactory and scientific position medical electricity and its cognate subjects. We have been favoured with copies of the syllabus of lectures for the term, and also with the questions set at the examinations which concluded the first term's work. From these it is evident that a useful training is to be obtained at the college. The value of the certificates given to the successful students is clearly enhanced by the fact that independent examiners, in the persons of W. E. Sampner, D.Sc. (Lond.), and J. Russell Harris, M.D., M.R.C.S., have been called in to examine, the former on electricity and the latter on anatomy and physiology. Some free studentships are offered to trained nurses.

Through our upward pilgrimage
Larger, deeper lessons learning,
May we still in labours blest,
Never tire and never rest;
And with forces ever new,
Serve the holy and the true.

—*Dean Stanley.*

### Everybody's Opinion.

[*Correspondence on all subjects is invited, but we cannot in any way be responsible for the opinions expressed by our correspondents. No communications can be entertained if the name and address of the correspondent is not given, or unless one side of the paper only be written on.*]

### PENSIONS FOR SERVANTS.

"A DISTRICT SUPERINTENDENT" writes: Many of your readers, like myself, must have been very glad indeed to hear of the General Domestic Servants' Benevolent Institution, and the pension fund connected with it, which the Secretary kindly brought to our notice in your issue of April 4th. Founded in 1846, the institution has, no doubt, been a great blessing, and long may it continue to be so. Its pension fund is a "benevolent fund," asking for just such payment from servants as shows their willingness to try to help themselves. There must, therefore, inevitably be uncertainty and delay in the granting of the pensions, obtained as they are by canvassing and election. Since 1846 our ideas of insurance, and of our powers of helping our poorer brothers and sisters to help themselves, have greatly developed. I cannot imagine a more perfect model of a pension fund than the "Royal National" for nurses. It adapts itself to all varieties of circumstances and dispositions. No provision now seems wanting, from the purely benevolent branch to the strictly commercial insurance for those who can afford the luxury of being perfectly independent (except in so far as they must owe a debt of gratitude to the originators and managers). It does not leave the day of sickness unprovided for, nor the contingency of members requiring to withdraw the money they have paid in. Is it too much to hope that the wise, philanthropic, and wealthy committee of the Servants' Benevolent Institution may see their way to evolving out of its present pension fund a wider scheme? We shall watch eagerly for the report of their annual meeting, to be held in St. James's Hall on May 5th.

### MALE NURSES.

"M." writes: I am glad to see that the male nurse question is being admitted to the columns of such a representative journal as THE HOSPITAL. The contribution which appeared in your issue of the 11th, by "One of Them," mentions a previous letter as to the training of male nurses in asylums, and he asks, Are male nurses and those who employ them satisfied with their training? He seems to disapprove of it, but gives no reason; we can guess why. I for one, who have had both hospital and asylum training, am quite satisfied that it is the training, knowledge, and tact acquired in asylums that is most essential to the male nurse. I will quote a passage from the article, where he says, "Men are absolutely necessary as nurses in special cases of male nursing." Just so, and for those cases a *special* knowledge is necessary, and in ninety-nine out of a hundred it is that which can be acquired *only* in asylums. The Military Hospital discipline in the ward, application of bandages to gunshot wounds, and ambulance work in the field are not likely to be much appreciated in civil life. Training in military hospitals is excellent in theory, but wretched in practice. I quite agree with "One of Them" as to the necessity of a certificate when trained, so that both nurses and the public may be protected from the imposition of every out-at-elbows valet, or second-hand warriors who choose to call themselves trained attendants. As to the uniform for male nurses the idea is absurd—altogether military. A practical man will know what suits him, and will wear it, and is not likely to hamper himself with a lot of unnecessary gear to drag about from place to place.

WALTER MAGUIRE writes: I have read with interest your articles on "Male Nurses," and I should like to know what thorough training your correspondent considers a male nurse should have before he is competent to take a case. We want no so-called training for men: leave that to female nurses; and it seems to me there is too much training and too little sound common sense now-a-days. Half the attendants from public institutions have to be trained afterwards for private nursing, therefore what good does the institutional experience do them? What's wanted in a male nurse is character. If he lacks this you may train him till he bursts, but you'll never make a successful nurse of him. The best male nurses I have ever met have been men "who wanted to know how to nurse," and if they knew anything kept it to themselves. The best trainer a male nurse can have is the medical man who employs him, and if he is loyal to the doctor his training will be wholesome and good. Again, if a male nurse has a lot of leisure time, let him find occupation for himself: read to or write for his patient, or mend his or his patient's clothing. Why not? Go on board some of Her Majesty's ships on a washing and mending day, and see Jack stitch and scrub; and are sailors effeminate? Idleness begets vice, and an idle man in a house, let him be a nurse or otherwise, soon becomes a nuisance. For uniform, nothing on duty is better than a good white linen jacket, as worn by some barbers; off duty, no uniform.

### THE PRIVATE NURSE.

NURSE LAURA writes: I am sorry the grievances of the private nurses are coming to the fore again, so much has already been said on the subject; but I think that we should also receive the statements of the nurse *cum grano salis*, for often she requires more attention than can reasonably be expected in a house of sickness. The comfort of the nurse depends chiefly upon herself, and when she is thoughtful and considerate to those for whom she is working, it is generally returned fourfold. I have been a private nurse for eighteen months, and with two exceptions, have always been treated with kindness and consideration; of course there are "Briars besetting every path, and a cross in every lot," and why should a private nurse expect her path to be all sunshine? I have always found the friends of my patients quite willing to conform to the rules of the institution to which I belong.

### Presentations.

MISS NOEMI ARMIT, Matron of the Bolton Infirmary, was presented on the 19th inst. by the sisters and nurses with a handsome travelling bag, with solid silver fittings, as an expression of their affection and regret at her resignation.

SISTER FROST and Miss Holditch were presented with a tea urn, brass gong, and a dozen dessert spoons, on the occasion of the anniversary of the opening of the Brassey Holiday Home.

### Notes and Queries.

TO CORRESPONDENTS.—1. Questions may be written on post-cards. 2. Advertisements in disguise are inadmissible. 3. In answering a query please quote the number. 4. If a private answer is desired, a stamped addressed envelope must be forwarded.

NOTICE.—We must really ask our correspondents to be more particular in enclosing name and address. Constantly we receive queries or letters with no name attached.

### Answers.

*E. S. S.*—Apply to the Matron, New Infirmary, Birmingham, or to St. Bartholomew's Hospital, London; but so much depends on the age, &c., of the would-be nurse that we cannot give full particulars here. See "The Hospital Annual."

*Dispensing.*—You can get lessons in dispensing at the Zenana Medical College, St. George's Road. S.W. (close to Victoria Station); or at 40, Charlotte Street, Portland Place, W.

*Nurse A. Perry.*—You are eligible to join the Royal National Pension Fund for Nurses, 8, King Street, Cheapside, E.C., for a pension; but you cannot join it for sick pay unless you belong to the Nurses' Club, or some central institution.

*A Matron.*—"Ministering Women" is a book, published in one volume and now ready. We cannot print your letter about "A Nurse in Trouble," because you have evidently forgotten the facts of the case.

*E.B.*—Parker's ward shoes are excellent. See advertisement.

*Mrs. Maitland.*—Get "The Englishwoman's Year Book," price 1s., published by Hatchards.

*S. P. Winchester.*—Miss Lückes had an article in the *New Review* last autumn dealing with the subject, and the Hospitals Association, 140, Strand, has issued some pamphlets dealing with the question. Mr. Rathbone's book on "District Nursing," and various articles which had appeared in our earlier numbers would give details.

*Nurse H.*—The contrivance for preventing children's ears from sticking out can be had from A. Claxton, 62, Strand, W.C.

*Ignoramus.*—Sulphonal has been too short a time in general use for any authoritative utterances to be made about its value, and the permanent consequences, if any, which follow its continued regular administration. What appears to be generally admitted is the following: That sulphonal is fairly potent as a sleep producer, though perhaps less sure in its action than chloral and morphia; that it produces its effects slowly; too slowly for convenience, to say the least; that so far as present experience goes, it seems pretty certain that very few observers have recorded serious after effects from the use of the drug. But it cannot be too steadfastly affirmed that the great probability is that all drugs which produce sleep by definite physiological action on the brain do, in the long run, tend to produce debility and disintegration of the cerebral structures; imbecility, in short, and what is called "softening of the brain." Many medical men may deny this, from an experience of half a-dozen cases. Such denial is of no scientific value whatever. The rule is that hypnotics, constantly used, injure the brain, and through it the whole body. Exceptions to rules there may always be, but when the rule takes the form of definite and constant physical sequences, from definite physical causes, the apparent exception is to be regarded with the keenest suspicion and the most careful scrutiny.

### The Invalid Children's Aid Association.

Part I.—WHAT THE ASSOCIATION DOES.

"I'll try and keep it clean, and I'll try and be a mother tc it," said a little sick girl, when asked what message she would send to a lady who had just given her a doll.

This poor child was suffering from curvature of the spine, and for eighteen months she had been lying upon her back, strapped down in a box splint. Her father was in a lunatic asylum and her mother was wretchedly poor. Their home was a single room on the third floor of a house in a back street, where the child lay, week after week and month after month, seeing nothing beyond the four walls of the room, with no toy but a broken doll, no book to read, or no knowledge of how to read it, if you had given her one. Truly, she had but few pleasures in her poor little life till the day when she was provided with a friend and visitor by the Invalid Children's Aid Association. Now, her visitor is teaching her to read, she is taken out in a spinal carriage to the park where she can see the trees and grass, she has plenty of toys to amuse her, including the doll, on whom she is prepared to bestow such maternal care. Best of all, she is taught to take an interest in other sick children, and her attention is turned from her own troubles by learning to sympathise with those of other people.

It is wonderful to see the interest the invalids take in one another.

"Please give this to the little girl, who is worse than I am, Nurse," said a little, paralysed girl, as she placed a doll, which she had dressed herself, in the hands of the sick nurse who visited her on behalf of the Association.

The poor child is so paralysed that she can use nothing but her hands; and now her great pleasure is to employ them in making as many different things as possible, first for one invalid she is told of, then for another, the last thing she made being a pretty holland scrap-book.

Some of the saddest cases are those of the "hip-children," the long-lying upon their backs becomes so inexpressibly wearisome, and they suffer so much pain from their terrible complaint. Often, for many a long month, their one cry is "my poor leg"; yet even then it is not difficult to interest them in someone else's "poor leg," and their own pain will grow less, as their attention is turned from themselves to others. This was particularly the case with a little Jewish child in East London, who was discharged from a hospital at the same time as another little invalid, both suffering from hip complaint. She was a wilful, impatient, little creature, who shrieked when she saw a doctor, before he had had time touch her; but she was full of concern for her fellow-sufferer, and deeply interested in the news which used to be brought to her by the Association nurse, who visited both the cases. On one occasion the nurse found her much troubled about an abscess on her leg, which had just broken, but it was not the pain she dwelt upon. She explained to the nurse that it was the disappointment she felt because the abscess broke, "and she did not hear it break."

The elder children seem generally to take it for granted that the little ones must be worse off than they are; however much they suffer themselves they seem to think it must be harder for the very young ones to bear pain. Then, too, they can let their sympathy take a practical form by working for the little ones, while it is not quite easy to set the little ones to work for their elders, though at the present time a little spinal child has just finished a wool-ball, which she began making many weeks ago, for a big girl in whom she was interested. Among the paralysed children, those whose speech is affected as well as their limbs are the most to be pitied.

One peculiarly sad case visited by the Association is that of an only child, whose mother is most of her time at work; the poor little thing has no companions, and the bed on which she lies looks out upon a brick wall. At one time the wall needed repair, and then she had the pleasure of watching the workmen, but that was an enjoyment which could not last for long, and the old blank would have come back again but for the Society, which found her out and befriended her. Now she has a big scrap-book to amuse her, and a doll to keep her company, and a visitor who can sometimes coax a smile on to her sad little face.

To provide a friend for every sick child who needs one is the first object of the Invalid Children's Aid Association. Further, it desires to strengthen the hands of such friends when extra help is needed, which the particular friend may be unable to give. Such help may take the form of surgical appliances, invalid carriages, skilled nursing where wounds have to be dressed or surgical instruments adjusted, admission to convalescent homes, change of air to the country or seaside, besides the giving of advice and information as to how the poor little invalids may be most efficiently relieved.

Such is a brief sketch of the aid given by the Association; next time I hope to say a few words as to how the Association itself can best be aided.

### Where to Go.

The next lecture will be given at the Midwives' Institute and Trained Nurses' Club on Friday evening, May 8th, at 7.45, by Dr. E. Muirhead Little. *Subject*: "Nursing of cases of Potts' Diseases of the Spine and some allied disorders." A few tickets to non-members 6d. each, for which early application must be made to the Secretary.

The Gresham Lectures on Astronomy will be delivered by the Rev. E. Ledger at Gresham College, Basinghall Street, at 6 p.m. on May 12th, 13th, 14th, and 15th. Mr. J. E. Nixon will deliver the lectures on Rhetoric on May 19th, 20th, 21st, and 22nd. Admission free.

The Silurian Amateur Dramatic Club will give a performance at Park Hall, Regent's Park, on May 21st, in aid of the Nurses' Club. Application for tickets should be made to Mrs. Nichol, Nurses' Club, 12, Buckingham Street, Strand, W.C.

### Amusements and Relaxation.

### SPECIAL NOTICE TO CORRESPONDENTS.

**Second Quarterly Word Competition commenced April 4th, ends June 27th, 1891.**

Competitors can enter for *all* quarterly competitions, but no competitor can take more than *one first prize* or *two* prizes of any kind during the year.

The words for dissection for this, the FIFTH week of the quarter, being

"MOLTKE."

| Names. | April 23rd. | Totals. | Names. | April 23rd. | Totals. |
|---|---|---|---|---|---|
| Christie | 22 | 118 | M. G. | 15 | 98 |
| Patience | 18 | 115 | Ivanhoe | — | — |
| Agamemnon | 20 | 118 | Weta | 19 | 93 |
| Hope | 23 | 120 | Lady Betty | 21 | 114 |
| Reldas | 19 | 115 | Mortal | 8 | 76 |
| Lightowlers | 18 | 113 | Little Eliza | 17 | 95 |
| Nurse J. S. | 12 | 79 | Dora | 20 | 93 |
| Qu'appelle | 21 | 114 | Ladybird | 18 | 92 |
| Jenny Wren | 20 | 104 | Psyche | 20 | 91 |
| Wyameris | 19 | 114 | Ugug | 11 | 83 |
| Paignton | 19 | 95 | Harrie | 8 | 18 |
| Theta | 19 | 111 | Grannie | 18 | 86 |
| Success | — | 17 | Eale | 15 | 78 |
| Tired | 17 | 91 | Grimalkin | — | 53 |

For Rules see The Hospital, April 4th, 1891.

### Notice to Correspondents.

N.B.—Each paper must be signed by the author with his or her real name and address. A *nom de plume* may be added if the writer does not desire to be referred to by us by his real name. In the case of all prize-winners, however, the real name and address will be published.

## Figures and Tables

**Figure f1:**
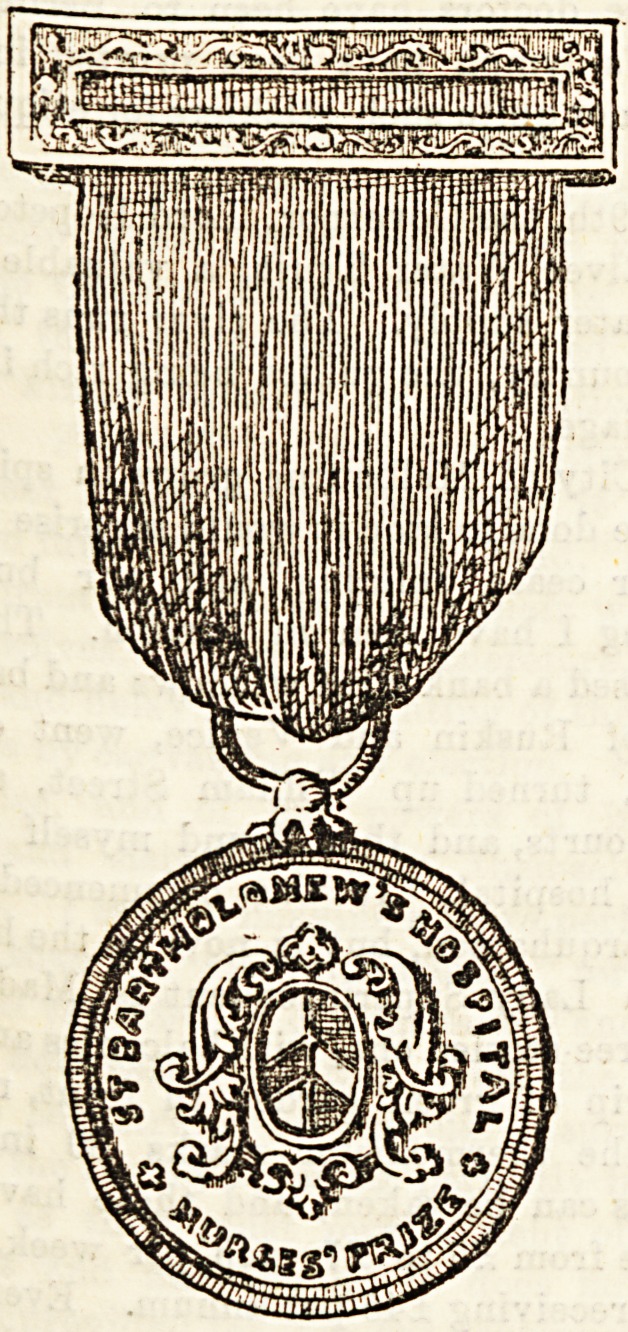


**Figure f2:**